# Development of KASP Markers and Identification of a QTL Underlying Powdery Mildew Resistance in Melon (*Cucumis melo* L.) by Bulked Segregant Analysis and RNA-Seq

**DOI:** 10.3389/fpls.2020.593207

**Published:** 2021-02-05

**Authors:** Yanyan Cao, Qiannan Diao, Youyuan Chen, Haijun Jin, Yongping Zhang, Hongmei Zhang

**Affiliations:** Shanghai Key Lab of Protected Horticultural Technology, Horticultural Research Institute, Shanghai Academy of Agricultural Sciences, Shanghai, China

**Keywords:** bulked segregant analysis, KASP markers, melon, powdery mildew, resistance gene

## Abstract

Powdery mildew (PM), caused by *Podosphaera xanthii* (*Px*), is one of the most devastating fungal diseases of melon worldwide. The use of resistant cultivars is considered to be the best and most effective approach to control this disease. In this study, an F_2_ segregating population derived from a cross between a resistant (wm-6) and a susceptible cultivar (12D-1) of melon was used to map major powdery mildew resistance genes using bulked segregant analysis (BSA), in combination with next-generation sequencing (NGS). A novel quantitative trait locus (QTL) named *qCmPMR-12* for resistance to PM on chromosome 12 was identified, which ranged from 22.0 Mb to 22.9 Mb. RNA-Seq analysis indicated that the *MELO3C002434* gene encoding an ankyrin repeat-containing protein was considered to be the most likely candidate gene that was associated with resistance to PM. Moreover, 15 polymorphic SNPs around the target area were successfully converted to Kompetitive Allele-Specific PCR (KASP) markers (*P* < 0.0001). The novel QTL and candidate gene identified from this study provide insights into the genetic mechanism of PM resistance in melon, and the tightly linked KASP markers developed in this research can be used for marker-assisted selection (MAS) to improve powdery mildew resistance in melon breeding programs.

## Introduction

Melon (*Cucumis melo* L.), which belongs to the Cucurbitaceae family, is an important horticultural and economic crop worldwide (Garcia-Mas et al., 2012). In view of its unique biological characteristics, such as a sweet fruit, unique aroma and rich nutritional value, melon is highly favored by consumers. Powdery mildew (PM) is a fungal disease that occurs commonly on leaves, petioles, and stems of most cucurbit crops in both field and greenhouse conditions (Perez-Garcia et al., 2009). This disease can result in a decrease in weight-based productivity and a reduction in fruit quality (Candido et al., 2014), thereby causing severe economic losses in many areas of the world (Romero et al., 2008).

Melon PM is often caused by *Podosphaera xanthii* (*Px*) and *Golovinomyces cichoracearum* (*Gc*) (Křístková et al., 2009; Li et al., 2017). These two fungi can be distinguished by observation of the pathogens’ cleistothecia, conidia germination, microscopic morphology, and host identification (Liang et al., 2010; Liu et al., 2010). Both pathogens exist either as an asexual stage (conidia) or sexual stage (Cleistothecia); the morphological characteristics of the asexual stage are similar for the two pathogens, and the morphological distinction between them is based on whether fibrous bodies occur on the spore (*Px* spores possess fibrous bodies, whereas these are absent from those of *Gc*) (Wang et al., 2013). In China, *Px* is generally considered to be the main causal agent of PM in melon (Cheng et al., 2011; Zhang et al., 2013). Recent reports have shown that melon PM is caused by *Px* in Shanghai (Gu et al., 2010; Li et al., 2015). The five phases of the *Px* life cycle are separately germinating conidia, the formation of a primary germ tube, hyphae, conidiophores, and colonization (Wang et al., 2013). After a spore lands on the leaf cuticle, the interaction is initiated by the formation of a primary germ tube and is followed by the formation and maturation of an appressiorial germ tube; the next step involves haustorium formation within an attacked epidermal cell and fungal development proceeds via the formation of secondary hyphae and haustoria and terminates with sporulation (Wolter et al., 1993). *Px* can coexist with the diseased plants in the soil as hyphae, conidia and cleistothecia, or it can overwinter as hyphae on greenhouse plants, and when the temperature rises the subsequent year, the conidia are distributed by air or water to infect the plants (Zhang et al., 2007).

Currently, the main method applied to control powdery mildew is chemical prevention by the application of chemical fungicides, which is often time-consuming and labor-intensive (Chen, 2014). Furthermore, chemical control not only leads to the appearance of resistance and to mutations in PM, which therefore means that this treatment becomes ineffective, but it also increases the input costs and has a negative impact on the environment (McGrath, 2001; Hollomon et al., 2002). Therefore, breeding for disease resistance is a safe alternative or a complement to the chemical control of this disease (Perchepied et al., 2005). Marker-assisted selection (MAS) is a powerful genomic tool that assists phenotypic selection for the development of disease-resistant cultivars and can help breeders incorporate and pyramid resistance genes into breeding material, thereby reducing disease severity (Ribaut et al., 2002; Chen, 2013; Zhu et al., 2019). At present, MAS has been extensively applied to search for the molecular markers that are linked to a specific trait during the development of disease-resistant cultivars (Teixeira et al., 2008). To date, a variety of molecular markers have been developed, such as RFLPs, RAPDs, SSRs, InDels and SNPs, to detect allelic variation within different samples at the DNA level (Wang et al., 2015).

Bulked segregant analysis (BSA) is an important technique used to map quantitative trait loci (QTLs) and identify DNA markers. Compared with the traditional QTL mapping method, which is time-consuming and involves screening polymorphic markers and genotyping, BSA provides a convenient and rapid method with which to identify resistance genes by generating two DNA bulks with a contrasting target trait (Michelmore et al., 1991; Abe et al., 2012; Nie et al., 2015). Recently, due to the release of sequenced genomes and the significant reduction in the costs of next-generation sequencing (NGS), whole-genome resequencing has been coupled with BSA to map the genes of interest that are associated with a given phenotype. The combined application of BSA with NGS (BSA-Seq) has accelerated the identification of tightly linked markers for gene identification and QTL mapping (Zou et al., 2016). To date, BSA-Seq has been successfully used in mapping the traits of early flowering, flesh thickness and downy mildew resistance in cucumber (Lu et al., 2014; Xu et al., 2015; Win et al., 2017), cold tolerance and blast resistance in rice (Yang et al., 2013; Zheng et al., 2016), cotyledon color, and a high-sucrose and low-oil seed phenotype in soybean (Dobbels et al., 2017; Song et al., 2017).

At present, the availability of sequence information has facilitated the identification and development of single nucleotide polymorphism (SNP) markers, which have largely replaced simple sequence repeats (SSRs) as markers in crop species (Semagn et al., 2014). Because of the low assay cost, high genomic abundance, ease of documentation, locus specificity, co-dominant inheritance, the potential for high-throughput analysis, and relatively low genotyping error rates, the use of SNPs has emerged as a powerful approach for many genetic applications in areas such as germplasm characterization, quality control (QC) analysis, linkage mapping, linkage-based and linkage disequilibrium-based QTL mapping, allele mining, marker-assisted backcrossing (MABC), genomic selection (GS), and MAS (Rafalski, 2002; Schlotterer, 2004; Semagn et al., 2014). Kompetitive Allele-Specific PCR (KASP) is a high-throughput SNP genotyping platform. Due to its low cost and genotyping error rates, and its high reliability and reproducibility, KASP has evolved to become a global benchmark technology and has been widely used for genetic mapping and trait-specific marker development (He et al., 2014; Ertiro et al., 2015; Rasheed et al., 2016; Tan et al., 2017).

To date, several genes and QTLs that confer resistance to powdery mildew have been identified in melon, such as the genes of *Pm-w* from WMR 29 (Pitrat, 1991), *Pm-x* from PI 414723 and *Pm-y* from VA 435 (Périn et al., 2002), and *Pm-1* from the AF125*^*Pm*^*^–^*^1^* Cantalupensis Charentais-type breeding line (Teixeira et al., 2008), and the QTLs of *PmV*.*1* and *PmXII*.*1* from PI 124112 (Perchepied et al., 2005), *Pm-R* from TGR-1551 (Yustelisbona et al., 2011) and *BPm12*.*1* from MR-1 (Li et al., 2017). In previous studies, many of these genes and QTLs have been found on chromosomes 2, 4, 5, and 12 (Pitrat, 1991; Périn et al., 2002; Fukino et al., 2008; Zhang et al., 2013; Li et al., 2017). Differing views exist concerning the genetic basis of PM resistance in melon. Some studies have indicated that PM resistance in melon is controlled by a single dominant gene (Epinat et al., 1992; Zhang et al., 2008; Wang et al., 2016; Li et al., 2017), whereas other research has reported that it is controlled by a recessive gene (McCreight and Coffey, 2011), by two dominant genes (Clements, 2014), or by one dominant and one recessive gene (Sun et al., 2010; Yuste-Lisbona et al., 2010). Moreover, it is also reported that resistance to PM in melon is controlled by different sets of QTL (Perchepied et al., 2005).

The melon cultivars wm-6 and 12D-1 are both high-generation inbred lines developed by our group and our previous study has shown that the melon cultivar wm-6 is highly resistant to PM, whereas 12D-1 is highly susceptible (Data not shown). In this study, we obtained an F_2_ population from a cross between wm-6 (female) and 12D-1 (male). A major QTL that confers PM resistance on chromosome 12 was identified by BSA-Seq analysis, and a most likely candidate gene was predicted from RNA-Seq data in wm-6 melon. In addition, 15 suitable KASP markers were developed by the KASP SNP genotyping method. This will facilitate the cloning and functional validation of the candidate resistance gene and the linked markers will further provide a useful tool for MAS in melon breeding programs.

## Materials and Methods

### Plant Materials, Growth Conditions and Inoculation With Powdery Mildew Fungus

Two inbred lines, wm-6 (P_1_, resistant to PM) and 12D-1 (P_2_, susceptible to PM), were used as parental lines to generate F_1_ and F_2_ populations for the QTL mapping of PM resistance in melon (*Cucumis melo* L.). The P_1_, P_2_, F_1_ and F_2_ individuals were all placed in a culture room at a temperature of 25/20°C (day/night) with a photoperiod of 14 h light and relative humidity of 50–75%. All the seeds used in this study were provided by the Shanghai Academy of Agricultural Sciences.

The PM fungus (*P*. *xanthii*) used in this study was isolated from leaves of diseased Cucurbitaceae plants according to the method of Nie et al. (2015). The plants were grown on the experimental farm of the Shanghai Academy of Agricultural Sciences, and the PM fungus was maintained by infection of susceptible melon cultivar plants. When the three true seedling leaves of melon plants were fully expanded, the fungus was collected and suspended in sterile distilled water containing 0.01% Tween 20 and was then used to inoculate plants at a concentration of 1 × 10^6^ as previously described (Zhang et al., 2011).

### Disease Evaluation for Resistance to Powdery Mildew

Phenotyping for powdery mildew resistance of melon was performed according to Zhang et al. (2013) with some modifications at 12-d post-inoculation (dpi), and each infected leaf was analyzed individually. Briefly, the disease grade of powdery mildew was categorized on a scale of 0–5 as follows: Class 0, no infection; Class 1, infection of less than 30% of the leaf; Class 3, infection of less than 70% of the leaf; Class 5, infection of approximately the entire leaf and coverage with heavy sporulation. The disease severity index (DSI) was calculated from the disease-rating scale using the following formula: DSI = 100 × Σ[(disease grade × number of plants in that grade)/(Total number of plants × maximum disease grade)]. For the inheritance study, lines with a DSI ≤ 20 were considered resistant and lines with a DSI > 20 were considered susceptible. For the F_2_ population, the same protocol was followed to identify resistant and susceptible plants. The phenotype of the two parental lines was analyzed at least three independent experiments with >15 seedlings examined in each experiment. The DSI of the two parental lines and the F_1_ hybrid plants was separately measured three times for 20 seedlings in each measurement. The DSI of F_2_ generation was calculated based on the phenotype of 193 F_2_ plants. On the basis of the DSI scores, plants with a DSI of 0–1 were categorized as resistant, and those with a DSI of 3–5, as susceptible.

### RNA Extraction, Library Construction and Sequencing

For RNA-Seq analysis, the two parental lines wm-6 and 12D-1 were separately treated with water (mock) or PM fungus. After 3 days, the leaves of wm-6 and 12D-1 plants (named wm-6K, wm-6P, 12D-1K, 12D-1P, respectively) were harvested, immediately frozen in liquid nitrogen and stored at −80°C before RNA extraction.

Total RNA was extracted using the Trizol reagent kit (Invitrogen, Carlsbad, CA, United States) according to the manufacturer’s protocol. RNA quality was assessed on an Agilent 2100 Bioanalyzer (Agilent Technologies, Palo Alto, CA, United States) and analyzed using RNase-free agarose gel electrophoresis. Following total RNA extraction, eukaryotic mRNA was enriched by Oligo(dT) beads, whereas prokaryotic mRNA was enriched by removing rRNA with the Ribo-ZeroTM Magnetic Kit (Epicentre, Madison, WI, United States). The enriched mRNA was fragmented into short fragments using fragmentation buffer and reverse transcribed into cDNA with random primers. Second-strand cDNA was synthesized by DNA polymerase I, RNase H, dNTPs and buffer. The cDNA fragments were purified with a QiaQuick PCR extraction kit (Qiagen, Venlo, The Netherlands), and following end repair and the addition of poly(A), was ligated to Illumina sequencing adapters. The ligation products were selected according to size by agarose gel electrophoresis, amplified by PCR, and sequenced using Illumina HiSeq2500 by the Gene *Denovo* Biotechnology Company (Guangzhou, China).

### Genomic DNA Extraction, Library Construction for Bulked Segregant Analysis and Whole-Genome Resequencing

Young leaves from the two parental lines, and from the F_1_ and the F_2_ populations were collected, and total genomic DNA was extracted using the CTAB method (Doyle, 1991). For bulked segregant analysis, four DNA pools were constructed, consisting of two parent bulks and two F_2_ segregating bulks. The parent bulks were separately constructed from the female parent (wm-6) and male parent (12D-1), and the two F_2_ segregating bulks were separately constructed by mixing an equal amount of DNA extracted from 25 extremely resistant (R-bulk) and 25 susceptible (S-bulk) F_2_ plants. After the four sequencing libraries were prepared according to the standard protocol of Illumina, they were sequenced on an Illumina HisSeq platform (Illumina, San Diego, CA, United States). Short reads obtained from the four bulks were aligned against the melon reference genome sequence to obtain the consensus sequence using BWA software. SNP calling was performed with GATK tools. The heterozygous alleles in both parents were filtered out during the process. The raw sequence reads are deposited in the NCBI Sequence Read Archive (SRA; Accession number: PRJNA655764).

### Genetic Mapping

In this study, four methods (SNP-ratio, ED^4^, G value and LOD) were used to map QTLs that underlay resistance to PM. The SNP-ratio (resistant alleles/sensitive alleles) of the R-bulk and S-bulk were calculated as described by Soyk et al. (2017) and the SNP-ratio of the R-bulk was then divided by the SNP-ratio of the S-bulk and plotted across the genomic regions that showed ratio peaks, which indicate the possible existence of the QTLs. The read depth for each allele at segregating allelic SNPs in 500-kb sliding windows was summed using a 100-kb step increment. The Euclidean distance of each SNP (ED-SNP) was calculated as described by Hill et al. (2013) and the ED was the sum of 100 ED-SNP values within a window of 100 consecutive SNPs. The ED^4^ was calculated by raising ED to the fourth power. The G value averaged across neighboring SNPs was calculated according to Magwene et al. (2011). The LOD (logarithm of the odds) score was calculated as described by Zhang et al. (2019).

### Development and Analysis of KASP Markers

The polymorphic SNPs identified around the target regions that associated with powdery mildew resistance were converted into KASP markers using PolyMarker software^1^. For each SNP, two allele-specific forward primers and one common reverse primer based on the flanking sequences around the variant position (SNP) were designed using Primer 3 software. The polymorphic SNP primers were converted to KASP markers to test their ability to differentiate the polymorphism by genotyping the two parents, and the KASP markers were then verified with the entire F_2_ population. Each KASP reaction was carried out using a 3-μL reaction mixture consisting of 1.48 μL KASP 2 × reaction mix, 50 ng DNA template, 0.17 μM Hex forward primer, 0.17 μM FAM forward primer and 0.42 μM universal reverse primer. The cycling conditions were as follows: 94°C for 15 min followed by 10 touchdown cycles at 95°C for 20 s and 65°C for 60 s (dropping 0.8°C per cycle); after the final annealing temperature of 56°C was reached, 26 cycles were performed at 94°C for 20 s and at 57°C for 60 s. Thermocycling and fluorescence readings were performed on a Hydrocycler and PHERAstar of LGC SNPline platform. Genotyping data were viewed as a cluster plot by SNPviewer software supported from LGC Genomics^2^. The significance of the correlation coefficients between phenotype and genotype was determined with *t*-tests. Linkage groups were constructed using JoinMap 4.1.

## Results

### Evaluation of Resistance to Powdery Mildew in wm-6 × 12D-1

**FIGURE 1:**
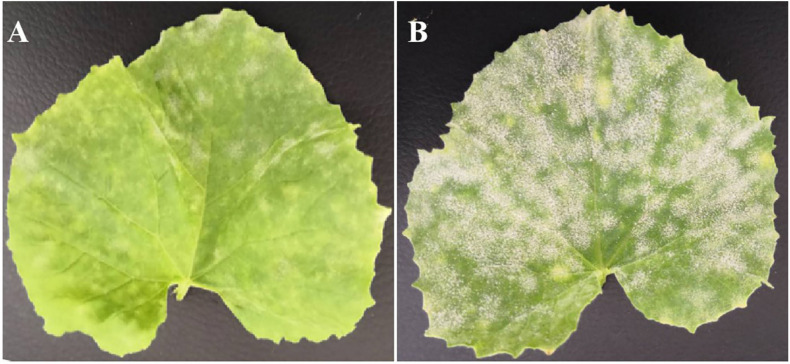
Phenotype of resistant parent wm-6 and susceptible parent 12D-1, 12 days after inoculation with *P*. *xanthii*. **(A)** The phenotype of the resistant parent wm-6, 12 days after inoculation with *P*. *xanthii*. **(B)** The phenotype of the resistant parent 12D-1, 12 days after inoculation with *P*. *xanthii*. The experiments were repeated at least three times independently, with similar results.

The artificial inoculation results showed that the parental line wm-6 was highly resistant to *P*. *xanthii* (DSI = 8.0), whereas the other parental line 12D-1 (DSI = 94.0) was susceptible to the fungus (Figure 1, Table 1 and Supplementary Table 1). The DSI of the F_1_ plants was 68.0, which meant that the F_1_ generation was susceptible to PM fungus (Table 1 and Supplementary Table 1). The evaluation of infection by PM indicated that 58 F_2_ plants showed PM resistance and 135 showed PM susceptibility, with a 1:3 segregation between resistant and susceptible individuals (χ*^2^* = 2.63, *P* = 0.11) (Table 1). This indicated that resistance to *P*. *xanthii* in wm-6 was conferred by a single recessive gene.

**TABLE 1:** Disease evaluation of powdery mildew in wm-6, 12D-1, F_1_ and F_2_ plants at 12 days post inoculation with *P*. *xanthii*.

Cross	Generation	Inoculation time	Disease rating^1^				Total	DSI
			0	1	3	5		
wm-6	P_1_	2019	12	8	0	0	20	8
12D-1	P_2_	2019	0	0	3	17	20	94
wm-6 × 12D-1	F_1_	2019	0	5	6	9	20	68
wm-6 × 12D-1	F_2_	2019	26	32	38	97	193	65.39

*The disease severity index of the two parental lines and the F_1_ hybrid plants were separately measured with 20 seedlings. The measurements were repeated three times independently with similar results, and the data of one representative experiment are shown. ^1^The DSI of leaves was rated on a 0–5 scale to determine the response of melon genotypes to powdery mildew, where 0 = immune, no symptom; 1 = highly resistant, infection of less than 30% of the leaf with low sporulation; 3 = moderately susceptible, infection of less than 70% of the leaf with moderate to high sporulation, and 5 = highly susceptible, infection of approximately the entire leaf with heavy sporulation.*

### Sequencing Data Analysis of Four DNA Bulks

BSA-Seq analysis was performed with the DNAs of four libraries (wm-6, 12D-1, R-bulk and S-bulk) using the Illumina HiSeq 2500 platform. In total, 60,401,327 and 57,755,318 clean reads for wm-6 and 12D-1, respectively, and 92,327,580 and 101,809,441 short reads for the R-bulk and S-bulk libraries, respectively, were generated. The GC content ranged from 36.76% to 37.1% and the Q20 and Q30 of each pool were over 98% and 93%, respectively. Over 95% of the reads were mapped to the melon reference genome, and the coverage rates were 95.78% in wm-6, 95.85% in 12D-1, 97.02% in R-bulk, and 97.05% in S-bulk, approximately resulting in a 23 × coverage depth for parental bulks and at least 35 × coverage for two F_2_ progeny bulks (Table 2). These results indicated that the quantity and quality of the data were sufficient for further analysis.

**TABLE 2:** Summary of sequencing data and the data aligned to the melon reference genome for the parental lines and the resistant and susceptible pools by BSA-Seq.

Sample name^1^	Clean reads^2^	Clean reads (%)	GC content (%)	Q20 (%)^3^	Q30 (%)^4^	Mapped reads (%)^5^	Coverage (%)^6^	Sequencing depth (×)
wm-6	60401327	95.86	36.80	98.02	93.17	95.80	95.78	23.34
12D-1	57755318	95.86	36.76	98.06	93.29	97.86	95.85	22.81
R-bulk	92327580	96.3	37.02	98.21	93.71	96.55	97.02	35.54
S-bulk	101809441	95.98	37.1	98.10	93.37	95.95	97.05	38.31

*^1^wm-6, powdery mildew-resistant parent; 12D-1, powdery mildew-susceptible parent; R-bulk, powdery mildew resistance bulk; S-bulk, powdery mildew susceptible bulk. ^2^Number of reads after trimming and adapter removal. ^3^The percentage of base recognition accuracy above 99%. ^4^The percentage of base recognition accuracy above 99.9%. ^5^Alignment to the melon genome assembly V4.0 (www.melonomics.net/). ^6^Coverage (≥1 read).*

### QTL Mapping of PM Resistance

**FIGURE 2:**
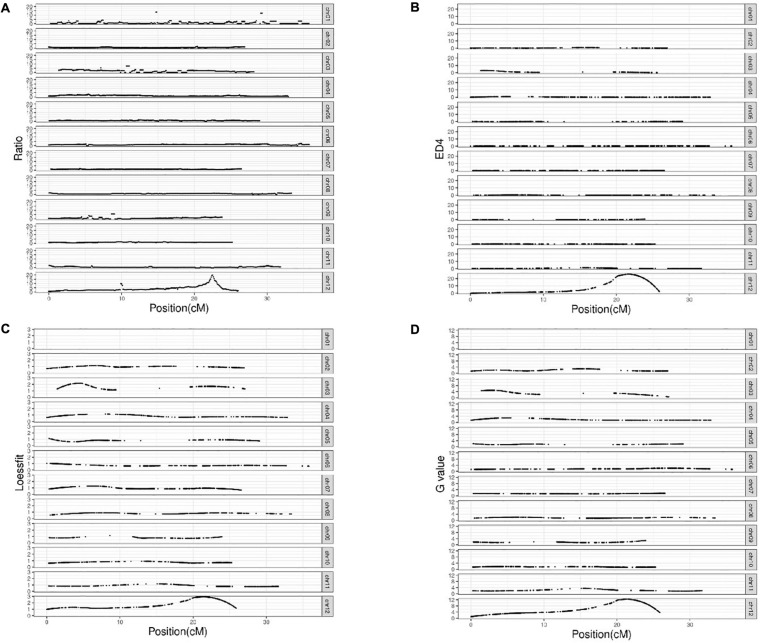
BSA-Seq results. Analysis of the QTL location by **(A)** the SNP-Ratio method; **(B)** the ED^4^ method; **(C)** the LOD method; **(D)** the G-value method.

After alignment to the reference genome of melon, 2,624,079 SNPs were identified between the parental lines wm-6 and 12D-1, and the distribution of these SNPs on each chromosome is listed in Supplementary Table 2. The resistance-related candidate regions were identified by analyzing the resistant pool and the susceptible pool. Using four statistic methods, a 0.9-Mb region spanning 22.0–22.9 Mb on chromosome 12 was defined as the target region associated with PM resistance (Figures 2A–D), and the significant QTL was designated *qCmPMR-12*. Within the candidate region, a total of 4,033 SNPs showed polymorphisms, 115 of which were located within the 3′ UTR, 78 in the 5′ UTR, 547 in the downstream region, 397 in the exonic region, 2,243 in the intergenic region, 1,005 in the intronic region, and 735 in the upstream region (Table 3). In addition, the target region included 182 non-synonymous SNV, 3 stopgain, 1 stoploss and 211 synonymous SNV polymorphisms (Table 4). Moreover, the candidate region contained 476 small InDels, 29 of which were located within the 3′ UTR, 23 in the 5′ UTR, 199 in the downstream region, 28 in the exonic region, 892 in the intergenic region, 387 in the intronic region, and 298 in the upstream region (Table 3). The InDels included 9 frameshift deletions, 6 frameshift insertions, 8 non-frameshift deletions and 5 non-frameshift insertions (Table 4).

**TABLE 3:** The chromosomal locations and numbers of SNPs and InDels in the target QTL region associated with powdery mildew resistance between the melon parental lines wm-6 and 12D-1.

Chromosomal location	3′ UTR	5′ UTR	Downstream	Exonic region	Intergenic region	Intronic region	Upstream
SNP number	115	78	547	397	2243	1005	735
InDel number	29	23	199	28	892	387	298

**TABLE 4:** The types and numbers of SNPs and InDels in the target QTL region associated with powdery mildew resistance between the melon parental lines wm-6 and 12D-1.

SNP type	SNP number	InDel type	InDel number
non-synonymous SNV^1^	182	frameshift deletion	9
stopgain	3	frameshift insertion	6
stoploss	1	non-frameshift deletion	8
synonymous SNV^1^	211	non-frameshift insertion	5

*^1^Single nucleotide variants.*

According to the melon gene annotation database^3^, the target region contains approximately 126 annotated genes, and the specific information for these genes is listed in Supplementary Table 3.

### RNA-Seq and Candidate Gene Analysis

To elucidate the changes in gene expression of resistant and susceptible melon lines after inoculation with *P*. *xanthii*, we performed an RNA-Seq analysis at 3 dpi. The dataset submitted to NCBI include the raw reads of the assembled transcriptome sequences from control and pathogen-treated melon plants. All transcriptome raw reads have been deposited in NCBI SRA^4^ under the accession number PRJNA670091.

Analysis of the RNA-Seq results for the 126 annotated genes obtained by BSA-Seq indicated that the transcript levels of the two genes *MELO3C002434* and *MELO3C002477* (| log_2_FC| > 1) were clearly up-regulated in the parental lines after inoculation with *P*. *xanthii*^5^. The two genes respectively encode an ankyrin repeat-containing (ANK) protein and a homeobox-leucine zipper protein (Supplementary Table 3). Further analysis showed that only the expression level of *MELO3C002434* was significantly higher in the resistant line wm-6 than in the susceptible line 12D-1 following PM infection. Notably, BSA-Seq showed that this gene contained 10 SNPs and 1 InDel (Table 5). Previous studies have shown that ANK proteins play important roles in regulating immune responses against various pathogens (Cao et al., 1997; Ryals et al., 1997; Yan et al., 2002; Li et al., 2013). Therefore, we speculated that *MELO3C002434* may confer PM resistance, or at least be a PM resistance-related gene in melon.

**TABLE 5:** Analysis of SNPs and InDels present within the *MELO3C002434* gene in two parental melon lines.

Type	Position	Reference	Allele	wm-6	12D-1
SNP	22665882	C	T	1/1	0/0
SNP	22666490	A	G	1/1	0/0
SNP	22666491	G	A	1/1	0/0
SNP	22666786	A	G	0/0	1/1
SNP	22666869	G	A	0/0	1/1
SNP	22667081	C	G	0/0	1/1
SNP	22667159	T	C	0/0	1/1
SNP	22668006	A	C	0/0	1/1
SNP	22668100	T	C	0/0	1/1
SNP	22668300	T	C	0/0	1/1
InDel	22666219	C	CATT	1/1	0/0

*0/0 means that the genotype of the inbred was the same as the reference, and 1/1 means that the genotype of the inbred was the same as the allele.*

### KASP Marker Development and Physical Map Construction

**FIGURE 3:**
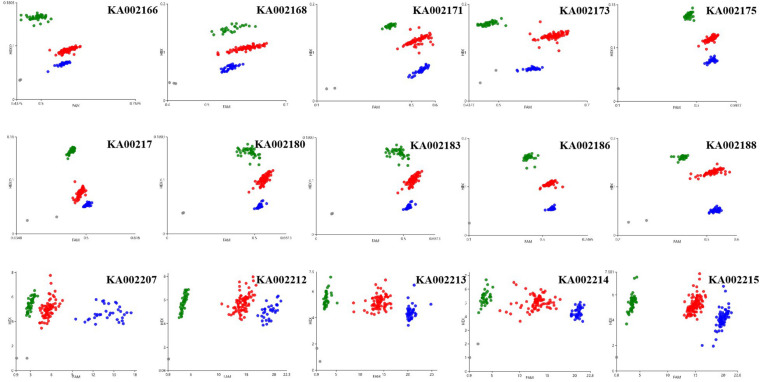
Results of genotyping using the Kompetitive Allele Specific PCR (KASP) assay. Scatter plots for selected KASP assays showing clustering of varieties on the X- (FAM) and Y- (HEX) axes. The green and blue dots represent the homozygous F_2_ lines and the red dot represents heterozygous F_2_ lines from the mapping population of the wm-6 × 12D-1 cross. The gray dots represent the NTC (non-template control).

**FIGURE 4:**
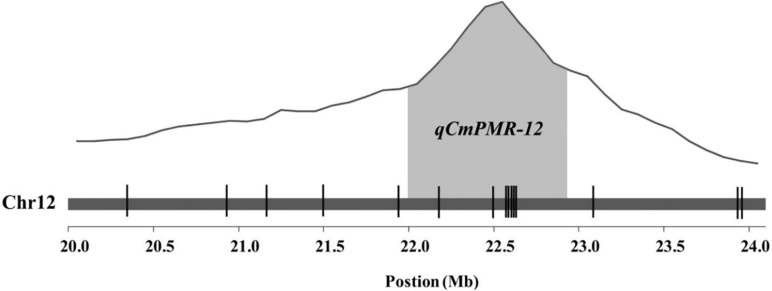
Quantitative trait locus (QTL) analysis of resistance to powdery mildew and the distribution of KASP markers linked to the target region. The gray areas represent the QTL region. The positions of KASP markers are shown by vertical lines.

KASP assays were designed for SNPs across the 20.0–23.9 Mb region on chromosome 12 and were tested on the F_2_ population to determine which markers showed the highest association with PM resistance. In this region, thirty-seven chromosome-specific SNPs selected for conversion to KASP markers were used to screen the parents and bulks to confirm their polymorphisms, and 15 out of 37 markers successfully distinguished the parents and bulks (Figure 3), indicating that these KASP markers were suitable for use in MAS to improve the level of powdery mildew resistance in melon breeding. Specific information and the sequences of the polymorphic KASP markers are separately listed in Supplementary Tables 4, 5. The close-up view of QTL and KASP markers linked to the target regions is shown in Figure 4.

**FIGURE 5:**
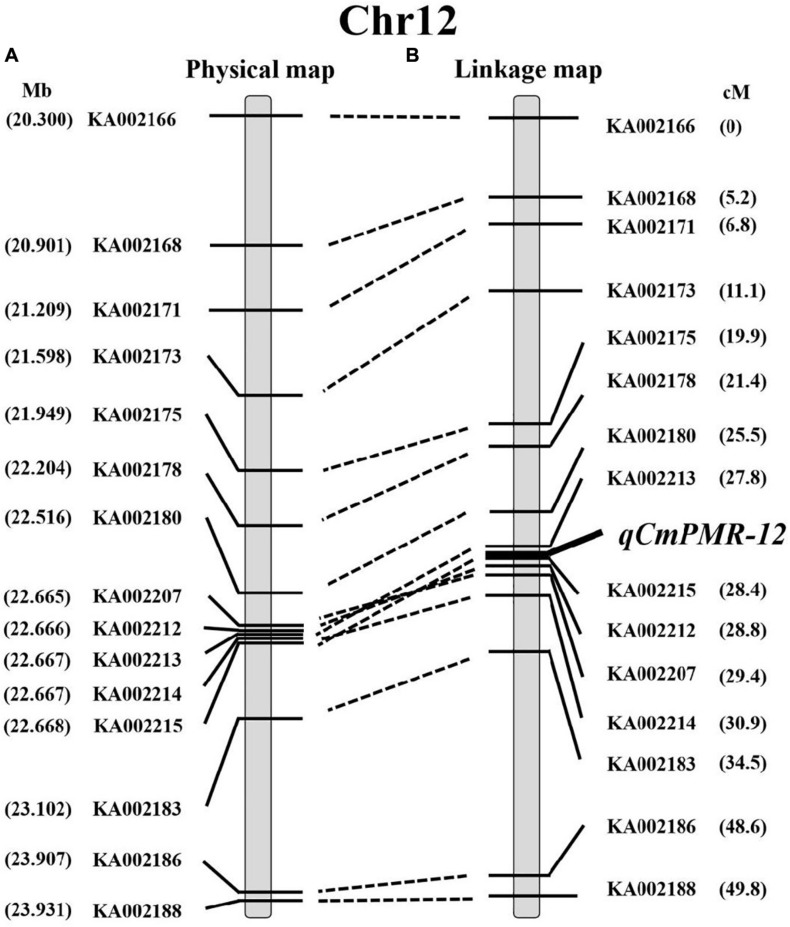
The location of *qCmPMR-12* on melon chromosome 12. **(A)** The physical map around the region of *qCmPMR-12*; marker names are indicated to the left of the map. **(B)** The genetic linkage map of melon chromosome 12; map distances are shown on the right. The QTL region was identified by QTL mapping using phenotypic analysis and marker data from F_2_ populations.

Using the 15 KASP markers, the genetic physical map and linkage map were constructed by JoinMap 4.1 (Figures 5A,B). As shown in the linkage map, the peak of the QTL was located in a 0.6-cM interval spanned by KASP markers KA002213 and KA002215 (Figure 5B).

## Discussion

The melon cultivar wm-6 is a high-generation inbred line developed by our group, which is highly resistant to powdery mildew (Figure 1A, Table 1 and Supplementary Table 1). In this study, a genomic region ranging from 22.0 Mb to 22.9 Mb on chromosome 12 was identified using BSA-Seq technology. Similarly, Li et al. (2017) detected a major QTL for PM resistance on chromosome 12 and suggested that resistance to *P*. *xanthii* in MR-1 was controlled by a single dominant gene. However, in this study, the segregation ratio of the F_2_ population indicated that the PM resistance in wm-6 was controlled by a single recessive gene, implying a different genetic basis of the PM resistance mechanisms between the melon cultivars wm-6 and MR-1. Other studies have also demonstrated that the genetic basis of resistance to PM differs depending on the tested melon material (Epinat et al., 1992; Zhang et al., 2008; Sun et al., 2010; Yuste-Lisbona et al., 2010; McCreight and Coffey, 2011; Wang et al., 2016).

To identify the PM resistance gene in wm-6, RNA-Seq analysis was performed with the melon lines wm-6 and 12D-1 in parallel. Combination of the results from BSA-Seq and RNA-Seq suggested that the *At3g12360*-like gene *MELO3C002434*, which encodes an ANK protein was the most likely candidate gene to confer PM resistance, because only this gene was significantly more highly expressed in the resistant line wm-6 than that in the susceptible line 12D-1 following infection with *P*. *xanthii* (see text footnote 5). Previous studies revealed that ANK proteins have critical functions in various biological processes of plant growth and development as well as in response to biotic and abiotic stresses (Albert et al., 1999; Yan et al., 2002; Ha et al., 2004; Hemsley et al., 2005; Garcion et al., 2006; Sakamoto et al., 2008; Shen et al., 2010; Li et al., 2013). It has been reported that the ANK protein NPR1 is important both in the SA-dependent immune response and in SA-independent resistance responses induced by the root-associated bacteria (Cao et al., 1997; Ryals et al., 1997); AKR2 functions in the oxidative metabolism of disease resistance and stress response in Arabidopsis (Yan et al., 2002); a plasma membrane-localized ANK protein, ACD6, is involved in SA-dependent signaling in defense responses and programmed cell death (Lu et al., 2003, 2005), and ectopic expression of the ANK protein *OsBIANK1* of rice confers enhanced disease resistance to *Botrytis cinerea* and *Pseudomonas syringae* in Arabidopsis (Li et al., 2013). Interestingly, the gene *MELO3C002434* contained 10 SNPs and 1 InDel (Table 5) and 5 out of 15 KASP markers that developed in this study were located within this gene (Supplementary Table 5). All of these results further implied that *MELO3C002434* is the most likely candidate gene associated with PM resistance in melon wm-6. In addition, the report of Li et al. (2017) suggested *MELO3C002434* to be one of the genes related to PM resistance in spite of the different genetic basis of the PM resistance mechanism in melon wm-6 and MR-1. Therefore, further genetic studies and more detailed analyses are required to confirm the role and molecular mechanism of action of *MELO3C002434* in the PM defense response.

In this study, 37 SNPs surrounding the candidate region were used to design KASP markers using the PolyMarker website and 15 of these were polymorphic among the bulk and parent populations. To the best of our knowledge, this is the first report that KASP markers have been developed and used in MAS to improve PM resistance in melon breeding, although several markers linked to resistance genes have been reported previously in melon (Ning et al., 2014; Han et al., 2015).

## Conclusion

In this study, a major QTL that is associated with PM resistance was identified in a 0.9-Mb interval on chromosome 12 of melon using BSA-Seq technology. Additional RNA-Seq data suggested that an ankyrin repeat-containing gene within this region, *MELO3C002434*, was implicated to be the most important candidate gene. In addition, 15 suitable KASP markers that were tightly linked to the resistance phenotype were developed for the MAS of melon. These data can be used to improve PM resistance in breeding programs and to facilitate understanding of the molecular mechanisms that underlying PM resistance in melon.

## Data Availability Statement

The raw sequence reads produced by BSA-Seq are deposited in the NCBI Sequence Read Archive (SRA; Accession number: PRJNA655764).

## Author Contributions

YZ, YC, and HZ designed the research. YC and QD prepared the plant materials. YC performed the experiments, analyzed the data, and wrote the manuscript. YZ, YC, and HJ revised the manuscript. All authors contributed to the article and approved the submitted version.

## Conflict of Interest

The authors declare that the research was conducted in the absence of any commercial or financial relationships that could be construed as a potential conflict of interest.

## Footnotes

1 http://polymarker.tgac.ac.uk

2 http://www.lgcgenomics.com

3 https://www.melonomics.net/

4 http://www.ncbi.nlm.nih.gov/sra

5 https://submit.ncbi.nlm.nih.gov/subs/sra/SUB8336313/

## Supplementary Material

Supplementary Table 1 Means, standard errors and ranges of DSI of the parents and F_1_ plants at 12 dpi with *P*. *xanthii* in the year of 2019.

Supplementary Table 2 The number of SNPs distributed on each chromosome of melon detected by BSA-Seq.

Supplementary Table 3 The positions and annotations of candidate genes in the target region conferring powdery mildew resistance from 22.0 Mb to 22.9 Mb based on the melon reference genome (CM3.6.1).

Supplementary Table 4 KASP primer sequences used for MAS in melon breeding programs.

Supplementary Table 5 Genotypes and positions of the 15 KASP markers linked to powdery mildew resistance gene of melon.
