# Supplementary material for: Development of KASP Markers and Identification of a QTL Underlying Powdery Mildew Resistance in Melon (Cucumis melo L.) by Bulked Segregant Analysis and RNA-Seq
Source: Front Plant Sci. 2021 Feb 5;11:593207. doi: 10.3389/fpls.2020.593207 (PMC7893098; doi:10.3389/fpls.2020.593207)
Supplement: Supplementary Table 1 — Means, standard errors and ranges of DSI of the parents and F1 plants at 12 dpi with P. xanthii in the year of 2019. [file Data_Sheet_1.zip › Supplementary Table 1.pdf]

Supplementary Table 1 Means, standard errors and ranges of the disease severity index of the parents and F_1 plants at 12 days post inoculation with *P. xanthii* in the year of 2019.

Cross	Generation	Inoculation time	Mean	Standard error	Range
wm-6	P_1	2019	9	0.82	8-10
12D-1	P_2	2019	88	4.32	84-94
wm-6 × 12D-1	F_1	2019	70	2.83	68-74

[1]days post-inoculation

[2]The Disease severity index

The DSI of leaves was rated on a 0 - 5 scale for determine the response of melon genotypes to powdery mildew, where 0 = immune, no symptom; 1 = highly resistant, infection of less than 30% of the leaf with low sporulation; 3 = moderately susceptible, infection of less than 70% of the leaf with moderate to high sporulation, and 5 = highly susceptible, infection of approximately the entire leaf and coverage with heavy sporulation.
