# Supplementary material for: Development of KASP Markers and Identification of a QTL Underlying Powdery Mildew Resistance in Melon (Cucumis melo L.) by Bulked Segregant Analysis and RNA-Seq
Source: Front Plant Sci. 2021 Feb 5;11:593207. doi: 10.3389/fpls.2020.593207 (PMC7893098; doi:10.3389/fpls.2020.593207)
Supplement: Supplementary Table 1 — Means, standard errors and ranges of DSI of the parents and F1 plants at 12 dpi with P. xanthii in the year of 2019. [file Data_Sheet_1.zip › Supplementary Table 2.pdf]

Supplementary Table 2 The number of SNPs distributed on each chromosome of melon detected by BSA-Seq.

Chromosome	ch01	ch02	ch03	ch04	ch05	ch06	ch07	ch08	ch09	ch10	ch11	ch12
SNP Number	23713	162002	248360	337684	222045	195620	280626	218290	136442	246364	328989	223944
