# Supplementary material for: Development of KASP Markers and Identification of a QTL Underlying Powdery Mildew Resistance in Melon (Cucumis melo L.) by Bulked Segregant Analysis and RNA-Seq
Source: Front Plant Sci. 2021 Feb 5;11:593207. doi: 10.3389/fpls.2020.593207 (PMC7893098; doi:10.3389/fpls.2020.593207)
Supplement: Supplementary Table 1 — Means, standard errors and ranges of DSI of the parents and F1 plants at 12 dpi with P. xanthii in the year of 2019. [file Data_Sheet_1.zip › Supplementary Table 3.pdf]

**Supplementary Table 3** The positions and annotations of 126 candidate genes in the target region conferring powdery mildew resistance from 22.0 Mb to 22.9 Mb based on the melon reference genome (CM3.6.1).

| Gene name | Start position | End position | Description of gene function |
|---|---|---|---|
| MELO3C000787.2.1 | 22103865 | 22105366 | - |
| MELO3C001571.2.1 | 22087405 | 22087642 | - |
| MELO3C002404.2.1 | 22885818 | 22887652 | zinc-binding protein [*Cucumis melo*] |
| MELO3C002405.2.1 | 22877123 | 22878043 | Putative 50S ribosomal protein L7/L12 [*Cucumis melo*] |
| MELO3C002406.2.1 | 22864303 | 22876338 | Putative bifunctional purine biosynthesis protein PurH [*Cucumis melo*] |
| MELO3C002407.2.1 | 22845384 | 22855707 | Mitogen-activated protein kinase kinase kinase 13-A [*Morus notabilis*] |
| MELO3C002408.2.1 | 22839730 | 22845271 | Putative potassium transporter 11-like isoform X1 [*Cucumis melo*] |
| MELO3C002409.2.1 | 22831842 | 22838436 | Putative potassium transporter 11-like [*Cucumis melo*] |
| MELO3C002411.2.1 | 22810795 | 22812174 | Polyadenylate-binding protein 1-B-binding protein [*Theobroma cacao*] |
| MELO3C002412.2.1 | 22804417 | 22809295 | Putative ankyrin repeat-containing protein At3g12360-like isoform X1 [*Cucumis sativus*] |
| MELO3C002413.2.1 | 22800418 | 22801306 | Putative ankyrin repeat-containing protein At5g02620-like [*Cucumis sativus*] |
| MELO3C002414.2.1 | 22791218 | 22795771 | Putative ankyrin repeat-containing protein At5g02620-like isoform X1 [*Cucumis melo*] |
| MELO3C002416.2.1 | 22772772 | 22777349 | Putative ankyrin repeat-containing protein At5g02620-like isoform X2 [*Cucumis melo*] |
| MELO3C002417.2.1 | 22768878 | 22772394 | Putative ankyrin repeat-containing protein At3g12360-like [*Cucumis melo*] |
| MELO3C002418.2.1 | 22760682 | 22763434 | Putative ankyrin repeat-containing protein At3g12360-like isoform X2 [*Cucumis melo*] |
| MELO3C002419.2.1 | 22755434 | 22756277 | Putative dirigent protein 9-like [*Cucumis melo*] |
| MELO3C002420.2.1 | 22750317 | 22751246 | Putative protein ENL-like [*Cucumis sativus*] |
| MELO3C002421.2.1 | 22745786 | 22748588 | Putative caffeoylshikimate esterase-like [*Cucumis melo*] |

| | | | |
|---|---|---|---|
| MELO3C002422.2.1 | 22737905 | 22738831 | Putative stigma-specific STIG1-like protein 1 [*Cucumis melo*] |
| MELO3C002423.2.1 | 22732564 | 22733037 | Putative stigma-specific STIG1-like protein 1 [*Cucumis melo*] |
| MELO3C002424.2.1 | 22731197 | 22731649 | Putative ankyrin repeat-containing protein At3g12360-like isoform X2 [*Cucumis melo*] |
| MELO3C002425.2.1 | 22727488 | 22729706 | Putative ankyrin repeat-containing protein At5g02620-like [*Cucumis sativus*] |
| MELO3C002426.2.1 | 22715917 | 22720695 | Putative ankyrin repeat-containing protein At5g02620-like [*Cucumis melo*] |
| MELO3C002428.2.1 | 22703326 | 22707274 | Putative protein ENL-like [*Cucumis sativus*] |
| MELO3C002429.2.1 | 22691534 | 22695449 | Putative ankyrin repeat-containing protein At3g12360-like [*Cucumis melo*] |
| MELO3C002430.2.1 | 22682939 | 22685201 | Putative wall-associated receptor kinase-like 14 [*Glycine max*] |
| MELO3C002431.2.1 | 22681442 | 22683416 | Putative probable pectate lyase 16 isoform X1 [*Gossypium arboreum*] |
| MELO3C002433.2.1 | 22670666 | 22671305 | Putative stigma-specific STIG1-like protein 1 [*Cucumis melo*] |
| MELO3C002434.2.1 | 22665764 | 22668402 | Putative ankyrin repeat-containing protein At3g12360-like [*Cucumis melo*] |
| MELO3C002435.2.1 | 22657059 | 22659134 | Putative protein ENL-like [*Cucumis sativus*] |
| MELO3C002436.2.1 | 22652386 | 22653946 | p55 [Cucumis sativus] |
| MELO3C002437.2.1 | 22645929 | 22646706 | p55 [Cucumis sativus] |
| MELO3C002438.2.1 | 22638465 | 22640986 | Putative ankyrin repeat-containing protein At5g02620-like isoform X1 [*Cucumis melo*] |
| MELO3C002439.2.1 | 22624931 | 22629199 | Putative ankyrin repeat-containing protein At5g02620-like [*Cucumis melo*] |
| MELO3C002441.2.1 | 22612861 | 22617037 | Putative ankyrin repeat-containing protein At5g02620-like [*Cucumis melo*] |
| MELO3C002442.2.1 | 22602864 | 22608221 | Putative aspartic proteinase isoform X2 [*Cucumis melo*] |
| MELO3C002443.2.1 | 22601798 | 22603299 | Putative probable F-box protein At4g22030 [*Cucumis melo*] |
| MELO3C002444.2.1 | 22597046 | 22600444 | Putative aminomethyltransferase, mitochondrial [*Cucumis melo*] |
| MELO3C002445.2.1 | 22594774 | 22595720 | Putative putative glycine-rich cell wall structural protein 1 [*Cucumis melo*] |
| MELO3C002446.2.1 | 22587211 | 22587576 | Putative glycine-rich cell wall structural |

| | | | |
|---|---|---|---|
| | | | protein [*Cucumis melo*] |
| MELO3C002447.2.1 | 22581219 | 22585805 | Putative L-ascorbate oxidase homolog [*Cucumis melo*] |
| MELO3C002448.2.1 | 22577674 | 22580586 | Putative tyrosyl-DNA phosphodiesterase 2 [*Cucumis melo*] |
| MELO3C002449.2.1 | 22571647 | 22576476 | Putative glucan endo-1,3-beta-glucosidase 1 [*Cucumis melo*] |
| MELO3C002450.2.1 | 22567594 | 22570767 | Putative 5'-adenylylsulfate reductase 1, chloroplastic [*Cucumis melo*] |
| MELO3C002451.2.1 | 22562970 | 22565470 | Glyoxalase domain-containing protein [Cephalotus follicularis] |
| MELO3C002452.2.1 | 22560147 | 22562470 | Putative autophagy-related protein 8C [*Cucumis melo*] |
| MELO3C002453.2.1 | 22557022 | 22559448 | Putative probable beta-1,3-galactosyltransferase 2 isoform X1 [*Cucumis melo*] |
| MELO3C002454.2.1 | 22550550 | 22551633 | Senescence regulator [*Corchorus olitorius*] |
| MELO3C002455.2.1 | 22534768 | 22545635 | Putative starch synthase 3, chloroplastic/amyloplastic [*Cucumis melo*] |
| MELO3C002456.2.1 | 22531458 | 22533006 | Putative ATP synthase gamma chain, chloroplastic [*Cucumis melo*] |
| MELO3C002457.2.1 | 22519596 | 22521851 | Putative peroxidase 42 [*Cucumis melo*] |
| MELO3C002458.2.1 | 22516214 | 22519208 | Homer protein isoform 2 [*Theobroma cacao*] |
| MELO3C002459.2.1 | 22507520 | 22508612 | Putative uncharacterized protein LOC103485322 [*Cucumis melo*] |
| MELO3C002460.2.1 | 22492373 | 22493009 | DUF4228 domain-containing protein [*Cephalotus follicularis*] |
| MELO3C002461.2.1 | 22483273 | 22488233 | Putative uncharacterized GPI-anchored protein At1g61900 [*Cucumis melo*] |
| MELO3C002462.2.1 | 22482131 | 22482764 | hypothetical protein Csa_1G063510 [*Cucumis sativus*] |
| MELO3C002463.2.1 | 22475795 | 22479428 | Putative UPF0051 protein ABCI8, chloroplastic [*Cucumis melo*] |
| MELO3C002464.2.1 | 22470843 | 22474581 | Putative polyadenylate-binding protein RBP45-like isoform X4 [*Cucumis melo*] |
| MELO3C002465.2.1 | 22461783 | 22462841 | Putative 15.4 kDa class V heat shock protein [*Cucumis melo*] |
| MELO3C002466.2.1 | 22450729 | 22460278 | Putative LOW QUALITY PROTEIN: abnormal spindle-like |

| | | | |
|---|---|---|---|
| | | | microcephaly-associated protein homolog [*Cucumis melo*] |
| MELO3C002468.2.1 | 22444886 | 22449263 | Putative tobamovirus multiplication protein 1 [*Cucumis melo*] |
| MELO3C002469.2.1 | 22439204 | 22440175 | hypothetical protein Csa_1G063600 [Cucumis sativus] |
| MELO3C002470.2.1 | 22429890 | 22438323 | Putative phospholipase A I isoform X1 [*Cucumis melo*] |
| MELO3C002471.2.1 | 22424144 | 22428217 | Putative RNA pseudouridine synthase 6, chloroplastic isoform X1 [*Cucumis melo*] |
| MELO3C002473.2.1 | 22417989 | 22423022 | Putative RNA pseudouridine synthase 6, chloroplastic-like isoform X1 [*Cucumis melo*] |
| MELO3C002474.2.1 | 22414246 | 22418733 | Putative beta-glucosidase 47 [*Cucumis melo*] |
| MELO3C002475.2.1 | 22404881 | 22407479 | Putative tryptophan synthase beta chain 1, chloroplastic-like isoform X1 [*Cucumis melo*] |
| MELO3C002476.2.1 | 22399159 | 22402036 | Putative tryptophan synthase beta chain 1-like [*Cucumis melo*] |
| MELO3C002477.2.1 | 22385995 | 22393830 | Putative homeobox-leucine zipper protein PROTODERMAL FACTOR 2 [*Cucumis melo*] |
| MELO3C002478.2.1 | 22377214 | 22377607 | Putative CRIB domain-containing protein RIC10-like [*Cucumis melo*] |
| MELO3C002479.2.1 | 22370138 | 22371622 | Putative nuclear transport factor 2 [*Cucumis melo*] |
| MELO3C002480.2.1 | 22365305 | 22367404 | Putative probable xyloglucan endotransglucosylase/hydrolase protein 8 [*Cucumis melo*] |
| MELO3C002482.2.1 | 22357405 | 22361820 | Putative chaperone protein dnaJ 50 [*Cucumis melo*] |
| MELO3C002483.2.1 | 22346578 | 22351676 | Putative gibberellin 2-beta-dioxygenase 8 isoform X2 [*Cucumis melo*] |
| MELO3C002484.2.1 | 22341979 | 22345745 | Putative probable pyruvate, phosphate dikinase regulatory protein, chloroplastic [*Cucumis melo*] |
| MELO3C002485.2.1 | 22340428 | 22343124 | Putative uncharacterized protein LOC103485579 [*Cucumis melo*] |
| MELO3C002486.2.1 | 22330427 | 22331546 | Putative NDR1/HIN1-like protein 12 [*Cucumis melo*] |
| MELO3C002488.2.1 | 22317779 | 22319547 | 40S ribosomal protein S9-2 [*Morus* |

| | | | |
|---|---|---|---|
| | | | *notabilis*] |
| MELO3C002489.2.1 | 22307652 | 22316501 | Putative cysteine-rich receptor-like protein kinase 10 [*Cucumis melo*] |
| MELO3C002491.2.1 | 22303171 | 22304403 | Putative cysteine-rich receptor-like protein kinase 25, partial [*Cucumis sativus*] |
| MELO3C002492.2.1 | 22299334 | 22300624 | Putative cysteine-rich receptor-like protein kinase 25 [*Cucumis melo*] |
| MELO3C002493.2.1 | 22295906 | 22299568 | aminoacyl-tRNA ligase [*Citrus limon*] |
| MELO3C002495.2.1 | 22290333 | 22293152 | Putative cysteine-rich receptor-like protein kinase 29 [*Cucumis melo*] |
| MELO3C002496.2.1 | 22280983 | 22289149 | Putative cysteine-rich receptor-like protein kinase 29 [*Cucumis melo*] |
| MELO3C002499.2.1 | 22277737 | 22278519 | Putative cysteine-rich repeat secretory protein 38-like [*Cucumis melo*] |
| MELO3C002500.2.1 | 22267088 | 22270293 | Putative uncharacterized protein LOC103485656 [*Cucumis melo*] |
| MELO3C002501.2.1 | 22262412 | 22265400 | Putative cysteine-rich receptor-like protein kinase 27 isoform X4 [*Cucumis melo*] |
| MELO3C002504.2.1 | 22242891 | 22252225 | Putative cysteine-rich receptor-like protein kinase 28 [*Cucumis melo*] |
| MELO3C002506.2.1 | 22229817 | 22238994 | Putative cysteine-rich receptor-like protein kinase 28 [*Cucumis melo*] |
| MELO3C002507.2.1 | 22226137 | 22229607 | Putative transcription factor ILR3-like [*Cucumis melo*] |
| MELO3C002508.2.1 | 22223159 | 22225386 | Putative thioredoxin-like protein CXXS1 [*Cucumis melo*] |
| MELO3C002509.2.1 | 22217024 | 22222349 | Putative kinesin-1 [*Cucumis melo*] |
| MELO3C002510.2.1 | 22211483 | 22213022 | Putative oxygen-evolving enhancer protein 3-2, chloroplastic [*Cucumis melo*] |
| MELO3C002511.2.1 | 22210074 | 22211444 | Putative uncharacterized protein LOC103485708 [*Cucumis melo*] |
| MELO3C002512.2.1 | 22207646 | 22209486 | Putative exopolygalacturonase-like [*Cucumis melo*] |
| MELO3C002513.2.1 | 22203933 | 22206026 | Putative protein HEAT-STRESS-ASSOCIATED 32 [*Cucumis melo*] |
| MELO3C002514.2.1 | 22197062 | 22199504 | Putative BTB/POZ domain-containing protein At3g22104-like [*Cucumis melo*] |
| MELO3C002515.2.1 | 22191857 | 22196696 | Putative uncharacterized protein LOC103485732 [*Cucumis melo*] |
| MELO3C002516.2.1 | 22165544 | 22169211 | Putative (3S,6E)-nerolidol synthase |

| | | | |
|---|---|---|---|
| | | | 1-like [*Cucumis melo*] |
| MELO3C002517.2.1 | 22140203 | 22146480 | Putative (3S,6E)-nerolidol synthase 1-like [*Cucumis melo*] |
| MELO3C002519.2.1 | 22117042 | 22117194 | - |
| MELO3C002520.2.1 | 22111113 | 22116528 | Putative (3S,6E)-nerolidol synthase 1-like [*Cucumis melo*] |
| MELO3C002521.2.1 | 22083111 | 22084488 | Putative uncharacterized protein LOC103485767 [*Cucumis melo*] |
| MELO3C002522.2.1 | 22069970 | 22073452 | Putative transcription factor bHLH112-like isoform X1 [*Cucumis melo*] |
| MELO3C002524.2.1 | 22054613 | 22067786 | Putative G-type lectin S-receptor-like serine/threonine-protein kinase At1g11410 [*Cucumis sativus*] |
| MELO3C002525.2.1 | 22052938 | 22053114 | - |
| MELO3C002526.2.1 | 22040878 | 22045068 | Putative protein DETOXIFICATION 40-like isoform X1 [*Cucumis melo*] |
| MELO3C002527.2.1 | 22035451 | 22039235 | Putative G-type lectin S-receptor-like serine/threonine-protein kinase At4g27290 isoform X1 [*Cucumis melo*] |
| MELO3C002528.2.1 | 22033421 | 22033717 | kinesin [*Cucumis melo*] |
| MELO3C002529.2.1 | 22028998 | 22030122 | Putative G-type lectin S-receptor-like serine/threonine-protein kinase At4g27290 isoform X1 [*Cucumis sativus*] |
| MELO3C002530.2.1 | 22024773 | 22028148 | Putative G-type lectin S-receptor-like serine/threonine-protein kinase At4g27290 isoform X2 [*Cucumis melo*] |
| MELO3C002532.2.1 | 22016954 | 22020180 | Putative G-type lectin S-receptor-like serine/threonine-protein kinase At4g27290 isoform X1 [*Cucumis melo*] |
| MELO3C002534.2.1 | 22009954 | 22013765 | Putative G-type lectin S-receptor-like serine/threonine-protein kinase At4g27290 isoform X1 [*Cucumis melo*] |
| MELO3C002535.2.1 | 22003631 | 22007112 | Putative G-type lectin S-receptor-like serine/threonine-protein kinase At4g27290 isoform X1 [*Cucumis melo*] |
| MELO3C035526.2.1 | 22183967 | 22188504 | Putative (3S,6E)-nerolidol synthase 1-like [*Cucumis melo*] |
| MELO3C035528.2.1 | 22021676 | 22023722 | Putative G-type lectin S-receptor-like serine/threonine-protein kinase At4g27290 [*Cucumis sativus*] |
| MELO3C035529.2.1 | 22464069 | 22467763 | Putative polyadenylate-binding protein RBP45-like [*Cucumis melo*] |

| | | | |
|---|---|---|---|
| MELO3C035727.2.1 | 22017146 | 22018120 | - |
| MELO3C035728.2.1 | 22176464 | 22178188 | - |
| MELO3C035729.2.1 | 22240996 | 22242173 | Putative LOW QUALITY PROTEIN: cysteine-rich receptor-like protein kinase 29 [*Cucumis sativus*] |
| MELO3C035730.2.1 | 22321327 | 22322619 | Putative cysteine-rich receptor-like protein kinase 25 [*Cucumis sativus*] |
| MELO3C035731.2.1 | 22527817 | 22531388 | pentatricopeptide repeat-containing protein [*Cucumis melo*] |
| MELO3C035732.2.1 | 22759728 | 22760332 | Putative ankyrin repeat-containing protein At3g12360-like isoform X1 [*Cucumis melo*] |
| MELO3C035733.2.1 | 22799073 | 22800358 | Putative ankyrin repeat-containing protein At3g12360-like isoform X1 [*Cucumis sativus*] |
| MELO3C035734.2.1 | 22801802 | 22802622 | Putative ankyrin repeat-containing protein At5g02620-like [*Cucumis sativus*] |
| MELO3C035735.2.1 | 22856687 | 22861213 | Putative protein FAR-RED IMPAIRED RESPONSE 1 isoform X1 [*Cucumis melo*] |

“-” means that no information about the function of the gene is available.
