# Supplementary material for: Development of KASP Markers and Identification of a QTL Underlying Powdery Mildew Resistance in Melon (Cucumis melo L.) by Bulked Segregant Analysis and RNA-Seq
Source: Front Plant Sci. 2021 Feb 5;11:593207. doi: 10.3389/fpls.2020.593207 (PMC7893098; doi:10.3389/fpls.2020.593207)
Supplement: Supplementary Table 1 — Means, standard errors and ranges of DSI of the parents and F1 plants at 12 dpi with P. xanthii in the year of 2019. [file Data_Sheet_1.zip › Supplementary Table 4.pdf]

Supplementary Table 4 Kompetitive allele specific PCR primer sequences used for marker-assisted selection in melon breeding programs.

KASP assay	Primer type	Primer sequence (5'-3')
KA002166	FAM	GAAGGTGACCAAGTTCATGCTGTTACCTTCTGGAAGACCTACAATC
	VIC	GAAGGTCGGAGTCAACGGATTGTTACCTTCTGGAAGACCTACAATT
	Com	CCTATGGATCGATTGACTTTGGGA
KA002168	FAM	GAAGGTGACCAAGTTCATGCTGTTGATTGATGGAGGTGAGCTCT
	VIC	GAAGGTCGGAGTCAACGGATTGTTGATTGATGGAGGTGAGCTCC
	Com	AGTCTCAATCCAAACCAGTAGCAT
KA002171	FAM	GAAGGTGACCAAGTTCATGCTTGCTTGCTATTTCTTATCGAATAATTGTT
	VIC	GAAGGTCGGAGTCAACGGATTTGCTTGCTATTTCTTATCGAATAATTGTC
	Com	ACACATGGACATAGACATGCATTG
KA002173	FAM	GAAGGTGACCAAGTTCATGCTGTTCTGAGGATGTGAGTGTGGT
	VIC	GAAGGTCGGAGTCAACGGATTGTTCTGAGGATGTGAGTGTGGC
	Com	CAATTGCTCCCGATTTAAGAAGCT
KA002175	FAM	GAAGGTGACCAAGTTCATGCTCCTTAGGGAAGAGGACATACTTGC
	VIC	GAAGGTCGGAGTCAACGGATTCCTTAGGGAAGAGGACATACTTGT
	Com	AGCTGCTCTTTACATACGACTGTC
KA002178	FAM	GAAGGTGACCAAGTTCATGCTTATTCACACCATGGAACCCTTTCG
	VIC	GAAGGTCGGAGTCAACGGATTTATTCACACCATGGAACCCTTTCA
	Com	GTCAACTCAGAGATTATGGTGGGT
KA002180	FAM	GAAGGTGACCAAGTTCATGCTGTTATTGCACAAATGGCTGATGAG
	VIC	GAAGGTCGGAGTCAACGGATTGTTATTGCACAAATGGCTGATGAA
	Com	AGCAAGTTGGGAATCCATAACTCT

KA002183	FAM	GAAGGTGACCAAGTTCATGCTGACGGATACAGCAGATACTTCCCA
	VIC	GAAGGTCGGAGTCAACGGATTGACGGATACAGCAGATACTTCCCT
	Com	CTGAGCAAGCAAACGTTGGATTAT
KA002186	FAM	GAAGGTGACCAAGTTCATGCTCAGATTAGACAAGCAAATCCTTATCG
	VIC	GAAGGTCGGAGTCAACGGATTCAGATTAGACAAGCAAATCCTTATCC
	Com	AGGAAATTAGAGTCCTGTGGCAAT
KA002188	FAM	GAAGGTGACCAAGTTCATGCTAAGCTAAGGACTTATGGTTGATCT
	VIC	GAAGGTCGGAGTCAACGGATTAAGCTAAGGACTTATGGTTGATCG
	Com	TCGTGTATCTCGATTGCTCATGAT
KA002207	FAM	GAAGGTGACCAAGTTCATGCTGTTTGTGATCGAGAAGACGAAGG
	VIC	GAAGGTCGGAGTCAACGGATTGTTTGTGATCGAGAAGACGAAGA
	Com	ATATGGGAAGCTCAGTGTTGACTT
KA002212	FAM	GAAGGTGACCAAGTTCATGCTAGCTTTGCATTTACTAGCTAGAAAGG
	VIC	GAAGGTCGGAGTCAACGGATTAGCTTTGCATTTACTAGCTAGAAAGC
	Com	GCTTGTTGCTGCTGCCAATT
KA002213	FAM	GAAGGTGACCAAGTTCATGCTGAGGCAACGAGGATAAAGATGGA
	VIC	GAAGGTCGGAGTCAACGGATTGAGGCAACGAGGATAAAGATGGC
	Com	CAGAGCTGCAACATCTGATATGAC
KA002214	FAM	GAAGGTGACCAAGTTCATGCTCATAACGTGCCGTCAAAATTGATAA
	VIC	GAAGGTCGGAGTCAACGGATTCATAACGTGCCGTCAAAATTGATAG
	Com	GTCATATCAGATGTTGCAGCTCTG
KA002215	FAM	GAAGGTGACCAAGTTCATGCTGCAGTACTCATCCTTCCAATTGTT
	VIC	GAAGGTCGGAGTCAACGGATTGCAGTACTCATCCTTCCAATTGTC
	Com	CCGCATTGTATCCAGAAACAGTTT
