# Supplementary material for: Development of KASP Markers and Identification of a QTL Underlying Powdery Mildew Resistance in Melon (Cucumis melo L.) by Bulked Segregant Analysis and RNA-Seq
Source: Front Plant Sci. 2021 Feb 5;11:593207. doi: 10.3389/fpls.2020.593207 (PMC7893098; doi:10.3389/fpls.2020.593207)
Supplement: Supplementary Table 1 — Means, standard errors and ranges of DSI of the parents and F1 plants at 12 dpi with P. xanthii in the year of 2019. [file Data_Sheet_1.zip › Supplementary Table 5.pdf]

Supplementary Table 5 Genotypes and positions of the 15 Kompetitive allele specific PCR markers linked to powdery mildew resistance gene of melon.

KASP marker	Homozygous resistance genotype[1]	Heterozygous genotype[2]	Homozygous susceptible genotype[3]	Position (bp)	P value
KA002166	TT	TC	CC	20300941	< 0.0001
KA002168	TT	TC	CC	20901452	< 0.0001
KA002171	TT	TC	CC	21209326	< 0.0001
KA002173	CC	TC	TT	21598014	< 0.0001
KA002175	TT	TC	CC	21949462	< 0.0001
KA002178	AA	AG	GG	22203919	< 0.0001
KA002180	AA	AG	GG	22516330	< 0.0001
KA002183	TT	AT	AA	23102800	< 0.0001
KA002186	CC	CG	GG	23907360	< 0.0001
KA002188	GG	TG	TT	23930811	< 0.0001
KA002207	TT	TC	CC	22665882	< 0.0001
KA002212	GG	CG	CC	22667081	< 0.0001
KA002213	AA	AC	CC	22668006	< 0.0001
KA002214	TT	TC	CC	22668100	< 0.0001
KA002215	TT	TC	CC	22668300	< 0.0001

[1]in powdery mildew resistant line wm-6

[2]in F_1 hybrids

[3]in powdery mildew susceptible line 12D-1
